# Sex dimorphism in serum lipid dynamics after acute exhaustive exercise

**DOI:** 10.5114/biolsport.2026.158677

**Published:** 2026-03-30

**Authors:** Zhongxun Ren, Baile Wu, Chunxue Tang, Jiayu Qian, Fan Zihan, YanYan Zhang, Lijun Shi

**Affiliations:** 1 Department of Exercise Physiology, Beijing Sport University, Beijing, China; 2 Laboratory of Sports Stress and Adaptation of General Administration of Sport, Beijing Sport University, Beijing, China; 3 Key Laboratory of Sports and Physical Health of Ministry of Education, Beijing Sport University, Beijing, China

**Keywords:** Lipidomics, Sexual dimorphism, Acute exercise, Metabolomics, Lipid metabolism

## Abstract

This study aimed to characterize serum lipidomic sexual dimorphism following acute exhaustive exercise under stringently controlled conditions. Forty healthy adults (20 males, 20 females) performed a CPET to exhaustion, with objective termination criteria and females tested during their mid-luteal phase. Untargeted lipidomics was performed on serum at baseline and at multiple postexercise time points. Males presented a greater absolute $\dot{\text{V}}$O_2peak_ (45.05 ± 4.14 vs 36.80 ± 3.69 ml/kg/min, P < 0.001) but a comparable $\dot{\text{V}}$O_2peak_ when FFM was normalized (56.50 ± 5.65 vs 55.45 ± 5.82 ml/kgFFM^/^min, P = 0.567). This standardized exhaustion was validated by both sexes achieving similar, high-intensity termination criteria (P_RERpeak_ = 0.607, P_RPE_ = 0.176, P_HRpeak_ = 0.164). A total of 620 lipid species were identified via untargeted lipidomics. Sex-stratified analysis identified more significantly different lipids compared to pooled-sex analysis (male:179, female:288). Key sex-discriminating lipids included PE 36:4, PC 32:1, SM d18:1/25:0, BMP 34:1, and PC 36:4. Cluster analysis revealed three recovery patterns, with lipid distribution differing significantly by sex. This study demonstrates that distinct metabolic response patterns exist between sexes when standardized exhaustion is achieved. Sex-stratified analysis identified more sex-specific lipids and pinpointed five sex-discriminating lipid molecular markers. The study revealed three different lipid recovery patterns, establishing that identical physiological endpoints arise from distinct metabolic mechanisms and underscoring the importance of sex-stratified analysis in exercise metabolism research.

## INTRODUCTION

Decades of research have confirmed sex differences in exercise metabolism, with the consensus that females exhibit a greater capacity for fat oxidation, whereas males favor carbohydrates [1, 2]. Whether this sex-specific response persists during exhaustive exercise and its molecular characteristics have been constrained by experimental complexity, particularly the challenge of defining a comparable endpoint of exhaustion between sexes [3].

Previous studies have often been limited by methods focused on only a few circulating substrates [4]. There is a lack of continuous monitoring during postexercise recovery and exercise intensity standardization methods that are limited by inherent physiological differences [5, 6]. Furthermore, testing females in the low-estrogen follicular phase to avoid hormonal fluctuations may have systematically underestimated true sexual dimorphism [7]. Recently, our team’s comprehensive untargeted metabolomics study systematically profiled serum metabolic responses following standardized exhaustive exercise, revealing distinct metabolic sexual dimorphism, with hypoxanthine, sarcosine, and lysophospholipids were identified as key sex-discriminating metabolites [8]. Lipids comprised the largest fraction (26–45%) of sex-specific metabolic responses in that study, highlighting their central role in exercise-induced sexual dimorphism. Comprehensive lipidomic characterization, including the molecular structures and temporal dynamics of complex lipid species such as phosphatidylcholine (PC), sphingomyelin (SM), and triacylglycerol (TAG), remains unexplored. Given that the lipid metabolome represents the predominant energy source during aerobic exercise, detailed lipidomic profiling is essential to fully understand the molecular mechanisms underlying sex-dimorphic exercise metabolism [9].

Using untargeted lipidomics, we captured the complete dynamic trajectory of serum metabolic perturbations at multiple time points following exercise. We hypothesized that under an equivalent physiological challenge, sex differences would manifest not only as quantitative shifts in substrate utilization but also as qualitative changes involving distinct lipid classes, regulatory networks, and recovery trajectories [10–12]. Therefore, the aim of this study was to construct, for the first time, a molecular map of lipid sexual dimorphism following acute exhaustive exercise, thereby providing a molecular foundation to support the development of personalized exercise strategies.

## MATERIALS AND METHODS

### Human Participants

**FIG. 1:**
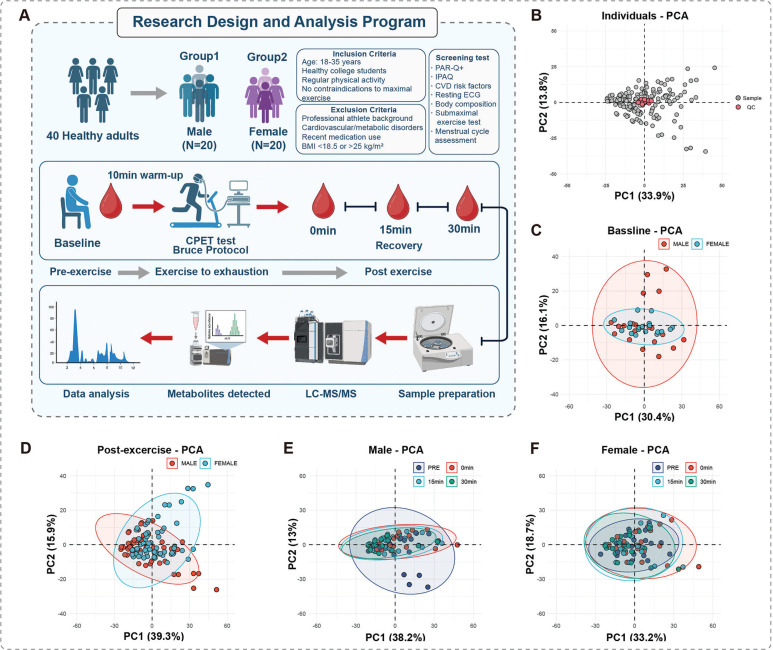
Study design and principal component analysis (PCA) of the serum lipidome. (A) Schematic of the experimental design. (B) PCA of all individual and QC samples. (C) PCA of baseline lipid profiles in males versus females. (D) PCA of combined postexercise lipid profiles in males versus females. (E, F) Temporal PCA of the exercise response in males (E) and females (F).

This cross-sectional study was designed to investigate the sex-specific serum lipid responses induced by acute exhaustive exercise. Forty healthy adults (20 males, 20 females) aged 18–35 years with a body mass index (BMI) between 18.0–25.0 kg/m^2^ were recruited from Beijing Sport University and the surrounding community through campus advertisements and social media (Figure 1A). The participant characteristics are detailed in Table 1. All participants completed screening questionnaires, including the PAR-Q+, the International Physical Activity Questionnaire (IPAQ), and a cardiovascular risk assessment. Body composition was assessed via dual-energy X-ray absorptiometry (DXA; GE Lunar iDXA, USA). The inclusion criteria were as follows: (1) healthy adults aged 18–35 years; (2) BMI between 18.0–25.0 kg/m^2^; (3) recreationally active (150–450 minutes of moderate-intensity exercise per week) according to the IPAQ assessment; (4) able to perform maximal intensity exercise safely; and (5) female with regular menstrual cycles (25–35 days). The exclusion criteria were as follows: (1) had cardiovascular disease or diabetes; (2) had metabolic, respiratory, or musculoskeletal disorders; (3) used prescription medications or supplements known to affect metabolism within the past month; (4) were pregnant, lactating, or had irregular menstrual cycles; and (5) smoked or consumed excessive alcohol (> 14 units/week). All female participants were tested during the mid-luteal phase, which was confirmed via a detailed menstrual history questionnaire and calendar-based tracking for at least two consecutive menstrual cycles prior to testing. This phase was chosen as sex hormone levels are elevated, to capture and study their intrinsic physiological state [13, 14]. Baseline variability was minimized by matching participants for age, body mass index (BMI), and $\dot{\text{V}}$O_2peak_ corrected for FFM.

The data are presented as the means ± SDs, with the range (minimum-maximum) provided in parentheses. Statistical comparisons between sexes were performed via independent samples t tests. BMI, body mass index; WHR, waist-to-hip ratio; $\dot{\text{V}}$O_2peak_, relative peak oxygen uptake; V_E_, minute ventilation; V_E_/$\dot{\text{V}}$O_2_, ventilatory equivalent for oxygen; V_E_/$\dot{\text{V}}$CO_2_, ventilatory equivalent for carbon dioxide; P_ET_O_2_, end-tidal oxygen tension; P_ET_CO_2_, end-tidal carbon dioxide tension; RR, respiratory rate; FFM, fat-free mass; HR, heart rate; RER, respiratory exchange ratio; RPE, rating of perceived exertion.

### Ethical approval and consent

The study protocol was approved by the Ethics Committee of Exercise Science Experiments, Beijing Sport University (No. 2024259H) and was registered in the Chinese Clinical Trial Registry (ChiCTR2400089036). The study adhered to the principles of the Declaration of Helsinki. All participants provided written informed consent after being fully informed of the study’s procedures and potential risks.

### Experimental Protocol and Pretest Standardization

To minimize confounding variables, participants were familiarized with the CPET equipment and RPE scale without physical practice. A standardized dinner (55% carbohydrate, 20% protein, 25% fat), with portions adjusted for individual energy needs (calculated via Mifflin-St Jeor and DXA), was consumed the evening before the test. For 72 hours, the participants refrained from strenuous activity, caffeine, and alcohol. All tests were conducted in a fasted state (10–12 hours overnight) between 07:00 and 10:00 to control for circadian rhythms in a temperature- and humidity-controlled laboratory (20–22°C, 40–60%).

### Cardiopulmonary Exercise Test Protocol

CPET was performed on a motorized treadmill following the standard Bruce graded exercise protocol [15]. Prior to the test, all participants completed a 10-minute standardized warm-up under the supervision of a professional. The Bruce protocol commenced at a speed of 1.7 mph (2.7 km/h) and a 10% grade, with speed and grade increasing synchronously every 3 minutes until the participant reached volitional exhaustion. During the test, gas exchange dynamics, including oxygen consumption ($\dot{\text{V}}$O_2_), carbon dioxide production (VCO_2_), and the RER, were continuously monitored and recorded via a highresolution metabolic gas analysis system (Cortex MetaLyzer^®^ 3B, Germany) [16]. HR was continuously recorded via a wireless HR monitor (Polar V800, Finland). Concurrently, the participants’ RPE was assessed every 2 minutes via the Borg 6–20 scale. All participants met pre-established maximality criteria [17]. These criteria included the following: (1) RER ≥ 1.10; (2) HR reaching > 90% of the age-predicted maximum (220 – age); (3) RPE ≥ 19; or (4) a plateau or decrease in $\dot{\text{V}}$O_2_ with increasing workload.

### Blood collection and sample preparation

Venous blood samples (5 mL each) were collected via separate venipunctures from an antecubital vein at four key time points: baseline (3–5 days prior to the exercise test), immediately postexercise (IP), 15 minutes postexercise, and 30 minutes postexercise. Upon cessation of the exercise protocol, participants were immediately assisted to a chair adjacent to the treadmill. Although defined as “immediately postexercise” (0 min), we acknowledge a brief logistical transition (movement to chair and venipuncture preparation). Strictly, all IP samples were collected within a 2-minute window following exercise cessation. Upon cessation of the exercise protocol, participants were immediately assisted to a chair adjacent to the treadmill, and the first postexercise sample was collected immediately in a seated position. The samples were collected in serum tubes, allowed to clot, and centrifuged at 1200 × g for 10 min at 4°C, after which the resulting serum was stored at -80°C. All the samples were processed within two hours of collection and were strictly limited to a single freeze–thaw cycle.

### Lipidomic analysis

Lipids were extracted from 50 µL of serum via a modified Bligh and Dyer method [18]. The lipid extracts were dried and stored at -80°C. Lipidomic analyzes were conducted at LipidALL Technologies via Jasper HPLC coupled with a Sciex TRIPLE QUAD 4500 MD mass spectrometer, as previously described [19]. Lipid classes were separated by normal-phase HPLC, and individual lipid species were quantified via MRM transitions by referencing a comprehensive panel of spiked internal standards (from Avanti Polar Lipids, Matreya LLC, Sigma–Aldrich, and CDN isotopes).

### Statistical analysis and data visualization

**FIG. 2:**
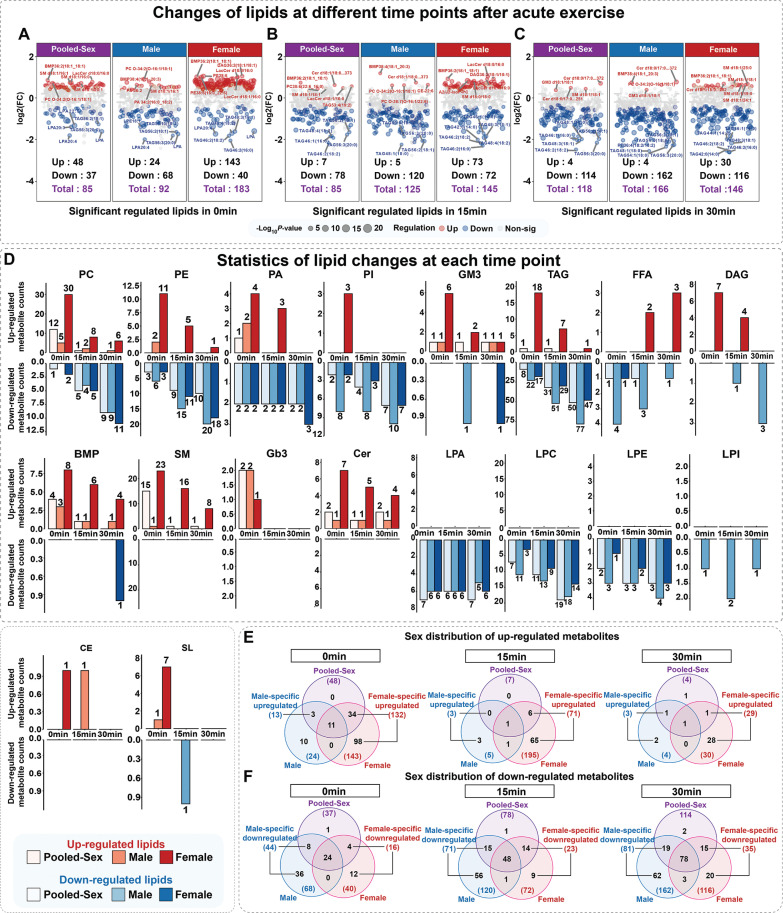
Overall characteristics and sex differences in serum lipids following acute exhaustive exercise. (A-C) Volcano plots of significantly altered lipids at 0 min (A), 15 min (B), and 30 min (C) postexercise. Analyzes are shown for the pooled cohort, males only, and females only. (D) Number of upregulated (red) and downregulated (blue) lipids across 18 major lipid classes at each postexercise time point. (E) Sex distribution of upregulated lipids. The Venn diagrams show the number of male specific, female specific, and shared upregulated lipids at 0, 15, and 30 minutes postexercise. (F) Sex distribution of downregulated lipids. The Venn diagrams show the number of male specific, female specific, and shared downregulated lipids at 0, 15, and 30 minutes postexercise.

The sample size was estimated via G-Power software (v3.1.9.7), which revealed that a minimum of 18 participants per group was required on the basis of a medium effect size (Cohen’s d = 0.6), 80% statistical power, and an α = 0.05. All lipidomics data were processed via R software (v4.4.3), and figures were created with Adobe Illustrator 2025 (v29.1). After quantile normalization and scaling, lipids were filtered on the basis of the detection rate (> 80%), CV in quality control (QC) samples (< 30%), and S/N ratio (> 3). Missing values (< 20%) were imputed via the k-nearest neighbors algorithm. Data normality was assessed by the Shapiro–Wilk test, and nonnormally distributed data were analyzed with corresponding nonparametric tests. All reported *P* values were adjusted for multiple comparisons via the Benjamini–Hochberg method to control the false discovery rate (FDR) at α = 0.05, unless otherwise specified. Independent t tests were used to compare baseline characteristics between sexes (Table 1), and principal component analysis (PCA) was employed to visualize the overall metabolic profiles and sex differences (Figure 1). Paired t-tests revealed widespread lipid alterations, with significance determined by a dual threshold of adjusted *P* < 0.05 and |log2(fold change)| > 0.2 (Figure 2). Two-way repeated-measures ANOVA (factors: sex, time, sex*time interaction), combined with orthogonal partial least squares discriminant analysis (OPLS-DA) variable importance in projection (VIP) score and area under the curve (AUC) analyzes, identified key lipids with sex-dependent responses [20]. The fuzzy c-means clustering algorithm groups lipids into three clusters on the basis of their temporal patterns [21]. These clusters were characterized by lipid class composition and structural features (e.g., carbon chain length and number of double bonds) (Figure 4).

**TABLE 1:** Baseline participant characteristics

	Male (n = 20)	Female (n = 20)	Male Vs. Female (*P* value)
**Anthropometric Measures**			
Height (m)	1.76 ± 0.06	1.63 ± 0.05	< 0.001
Body mass (kg)	68.8 ± 8.4	58.0 ± 5.8	0.008
Age (years)	22.9 ± 3.8	21.9 ± 2.8	0.474
BMI (kg·m^−2^)	22.3 ± 1.7	22.5 ± 2.4	0.862

**Body Composition**			
Body fat (%)	20.1 ± 3.3	34.5 ± 3.0	< 0.001
Fat-Free Mass (kg)	54.8 ± 5.9	38.5 ± 3.0	< 0.001
WHR	0.82 ± 0.03	0.74 ± 0.04	< 0.001

**Cardiorespiratory Measures**			
$\dot{\text{V}}$O_2peak_ (ml/kg/min)	45.1 ± 4.1 (32–54)	36.8 ± 3.7 (31–43)	< 0.001
$\dot{\text{V}}$O_2peak_ (ml/kgFFM/min)	56.5 ± 5.7 (48.1–69.0)	55.5 ± 5.8 (47.0–61.5)	0.567
*V*_E_ Max (l/min)	128.6 ± 15.1 (110.8–150.9)	75.3 ± 10.4 (61–91.5)	< 0.001
*V*_E_/$\dot{\text{V}}$O_2_ Max	35.2 ± 5.4 (31.2–48.8)	32.8 ± 3.9 (26.1–37.7)	0.276
*V*_E_/$\dot{\text{V}}$CO_2_ Max	29.6 ± 3.2 (25.9–34.7)	29.0 ± 2.2 (24.6–32.3)	0.600
*P*_ET_O_2_ Max (mmHg)	117.7 ± 3.6 (114.4–121.8)	115.5 ± 3.4 (109.1–119.6)	0.181
*P*_ET_CO_2_ Max (mmHg)	37.5 ± 3.6 (36.8–43.2)	39.3 ± 3.3 (34.9–45.7)	0.285
RR Max	49 ± 8 (39–63)	45 ± 3 (42–51)	0.120
Resting HR (bpm)	73 ± 15 (42–84)	79 ± 12 (55–115)	0.248
HR_peak_ (bpm)	192 ± 9 (175–205)	187 ± 9 (163–203)	0.164
RER_peak_	1.26 ± 0.09 (1.13–1.50)	1.24 ± 0.10 (1.10–1.37)	0.607
Endpoint RPE	19.6 ± 0.5 (19–20)	19.8 ± 0.4 (19–20)	0.176

**Exercise Duration**			
Exercise time (sec)	744.4 ± 114.0	623.3 ± 97.1	0.008

## RESULTS

### Cohort baseline characteristics and data quality control

All participants (20 males, 20 females) performed the exercise protocol until volitional exhaustion without interruption. Both sexes met the criteria indicating achievement of maximal exercise, with no significant differences in physiological termination parameters (Table 1). Furthermore, no significant sex difference was observed in $\dot{\text{V}}$O_2peak_ after normalization to FFM (*P* = 0.567), further confirming that both groups endured a similar level of relative exercise intensity (Table 1). PCA was performed to visualize the overall distribution and characteristics of the samples. The tight clustering of all QC samples demonstrated the stability and reliability of the analytical platform. Figures 1C and 1D show the distribution and relationship between the sexes at baseline and postexercise, respectively. Furthermore, the intra-group analyzes for each sex (Figures 1E and 1F) clearly show that the postexercise samples as a whole did not show a clear separation from their baseline position, but individual differences were pronounced.

### Overall Characteristics and Sex Differences in Lipid Changes Driven by Acute Exercise

A total of 620 lipid species were identified and quantified via untargeted lipidomic analysis. Significantly altered lipids were defined as those exhibiting an adjusted *P* value < 0.05 and |log_2_FC| > 0.2 at any postexercise time point compared with baseline. A comparison of lipid levels at the three time points against baseline revealed that acute exhaustive exercise rapidly and persistently induced widespread perturbations in the serum lipid profile. In total, 367 lipids were significantly altered postexercise (male:179, female:288). By incorporating sex as a variable in the analysis, a greater number of significantly different lipids could be identified for both sexes (Figure 2A-C). The number of significantly different lipids in males and females at 0, 15, and 30 minutes postexercise was approximately 8.2%-115.3% higher than the results from the pooled-sex analysis. However, the number of significantly changed lipids over time showed a trend of decreasing upregulated lipids and increasing downregulated lipids. Chemical classification (Figure 2D) revealed that a total of 18 classes of lipids were found to be altered, exhibiting distinct sexual dimorphism. The Venn diagrams (Figure 2E, F) show that the number of male specific altered lipids increased over time, with TAG being the most significantly affected class (N_0min_ = 16, N_15min_ = 29, N_30min_ = 34). In contrast, the number of female specific altered lipids tended to decrease over time, with TAGs still being the most abundant altered lipid type (N_0min_ = 27, N_15min_ = 11, N_30min_ = 4). The specific identities of these sex-dependent altered lipids are detailed in Supplementary Table S1 (link).

### Differential Lipids Stimulated by Acute Exhaustive Exercise Exhibit Sexual Dimorphism

**FIG. 3:**
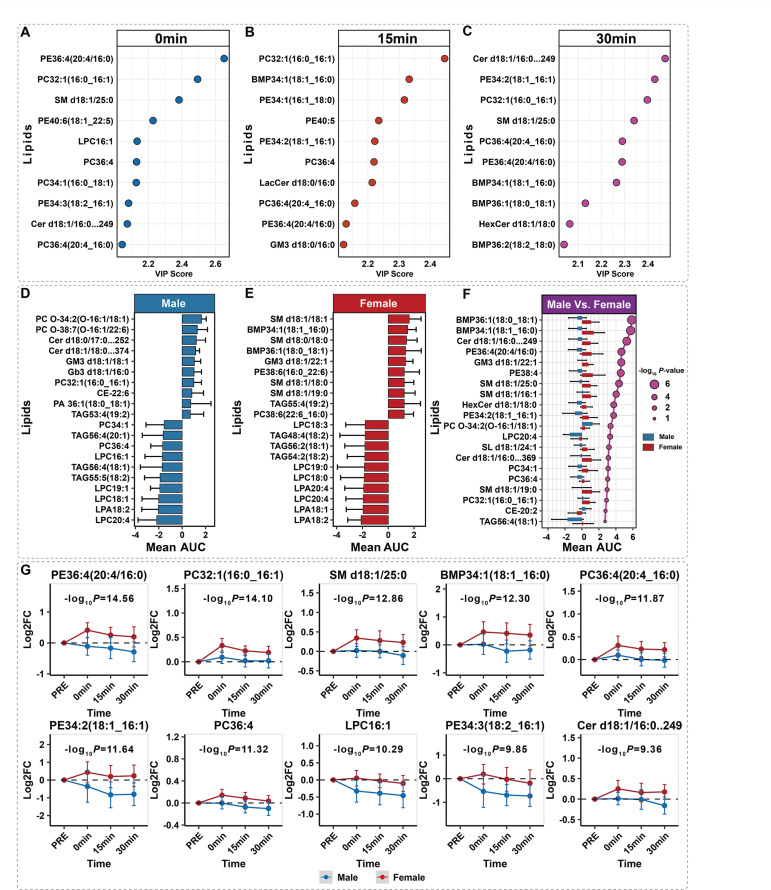
Identification of key sex-dimorphic lipids via a multistrategy analytical approach. (A-C) The top 10 lipids discriminating between sexes at 0, 15, and 30 min postexercise, as ranked by the OPLS-DA VIP score. (D, E) Top 10 lipids with the largest cumulative change in the mean AUC postexercise in males (D) and females (E). (F) The top 20 lipids with the most significant differences in the mean AUC between males and females, as determined via Log2 FC analysis. The direction of the bars indicates the sex with the highest cumulative response, while colors distinguish between sexes (blue for males, red for females). The size of the dots represents statistical significance (−Log10(P value)). (G) Time course of key lipids identified by two-way ANOVA, ranked by the significance of the sex effect.

We used a combined approach, employing OPLS-DA to identify lipids with the highest sex-discriminating power, AUC analysis to quantify lipids with the greatest cumulative changes, and two-way ANOVA to identify lipids with sex-differentiated dynamic patterns. The VIP analysis showed that phosphatidylethanolamine (PE) species PE 36:4(20:4/16:0) ranked high at all three time points (Figure 3A-C). The AUC analysis showed that in males, the lipids with the largest and smallest cumulative changes were PC species PC O-34:2(O-16:1/18:1) and lysophosphatidylcholine (LPC) species LPC20:4, respectively, while in females, they were SM species SM d18:1/18:1 and lysophosphatidic acid (LPA) species LPA18:2 (Figure 3D-F). The lipids with the most significant magnitude of difference in AUC were bis(monoacylglycerol)phosphate (BMP) species BMP36:1(18:0_18:1) and BMP34:1(18:1_16:0). Combining the results from VIP (ranking high at two or more time points), significant magnitude of AUC difference, and the sex effect from the two-way ANOVA consistently identified PE 36:4, PC 32:1, SM d18:1/25:0, BMP 34:1, and PC 36:4; these lipids are important indicators of the sex-differentiated response following exhaustive exercise.

### Dynamic patterns of the sex-specific lipidome

**FIG. 4:**
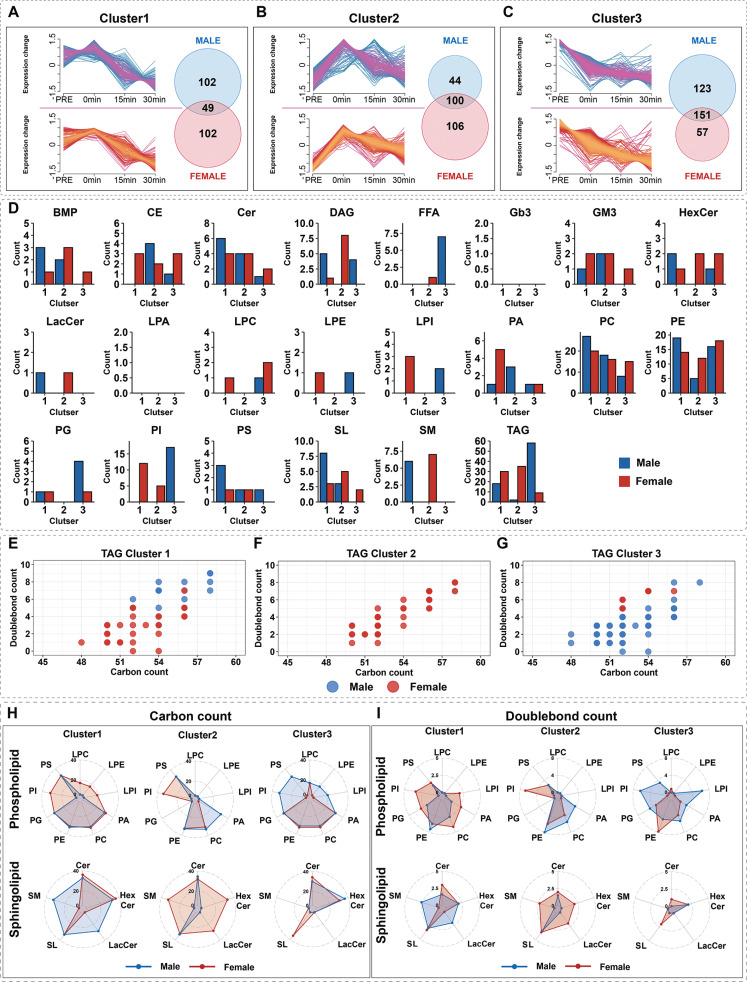
Cluster analysis of sex-specific lipid response patterns after acute exercise. (A-C) Three lipid response patterns identified by fuzzy c-means clustering. Left: Expression trends. Right: Venn diagrams of lipid distribution by sex. (D) Sex-specific distribution of major lipid classes within each cluster. The bar chart shows the number of different lipid classes in the three clusters, distinguished by sex (blue: male, red: female), revealing the enrichment of these classes in the different response patterns. (E-G) Structural properties (carbon number vs. number of double bonds) of the TAGs in each cluster. (H, I) Radar plots comparing the average carbon chain length (H) and double bond number (I) of phospholipids and sphingolipids between sexes across clusters.

Fuzzy c-means clustering grouped the responding lipids into three distinct temporal patterns. In the “Delayed Downregulation” pattern (Cluster 1) (Figure 4A, E), there was a comparable number of lipids from both sexes; male TAGs (C54-C58) had longer and more unsaturated carbon chains than female TAGs (C50-C54). The “Elevated then Recovered” pattern (Cluster 2) (Figure 4B, F) was female-dominant, and all TAGs in this cluster (C50-C58) were from females. The “sustained downregulation” pattern (Cluster 3) (Figure 4C, G) was male-dominant; male TAGs (C48-C54) had shorter and more saturated carbon chains than female TAGs (C52-C56). The radar plots (Figure 4H, I) show that in Cluster 1, female phosphatidylinositol (PI), LPC, lysophosphatidylethanolamine (LPE), and lysophosphatidylinositol (LPI) exhibited a unique high-unsaturation distribution, while in Cluster 2, PI was specifically enriched in females and phosphatidic acid (PA) was specifically enriched in males. Regarding sphingolipids, females in Cluster 1 exhibited longer-chain ceramide (Cer) and hexosylceramide (HexCer), while in Cluster 2, the specific sphingolipids SM and lactosylceramide (LacCer) had a higher number of double bonds in females. Male PI, phosphatidylserine (PS), LPE, and LPI showed a specific distribution; their phosphatidylglycerol (PG), PC, and PA had higher unsaturation than in females, while their PE and LPC had relatively lower unsaturation.

## DISCUSSION

This study, for the first time, presents a comprehensive, sex-differentiated lipid atlas of the serum metabolic response following acute exhaustive exercise in healthy young adults. The study design placed particular emphasis on the standardized control of the state of exhaustion by employing objective termination criteria (RER_peak_, HR_peak_, RPE). Crucially, this standardized physiological state was further validated by the lack of a significant difference in $\dot{\text{V}}$O_2peak_ after normalization to fat-free mass. This approach is supported by recent mechanistic studies demonstrating that normalizing to lean mass effectively eliminates sex differences in key central hemodynamic determinants [22]LBMs. Furthermore, when differences in lean mass are accounted for, the physiological responses to exhaustive exercise and the magnitude of functional reserve are remarkably similar between sexes [23]. Collectively, these findings confirm that our sexstratified cohorts were tested under a comparable physiological strain, ensuring that the observed lipidomic dimorphism reflects fundamental differences in metabolic regulation rather than disparities in relative fitness. Despite achieving this comparable state of exhaustion, males and females presented distinctly different metabolic characteristics (Table 1). This comprehensive analytical approach provides a robust and reliable methodological template for future research on sex-based differences in exercise metabolism.

Notably, our cohort comprises healthy young adults (18–34 years), which is a significant distinction from the general population in the SHIP cohort with a wider age coverage (20–79 years). The mean $\dot{\text{V}}$O_2peak_ of our participants (Male: 45.1 ± 4.1 ml/kg/min; Female: 36.8 ± 3.7 ml/kg/min) is substantially greater than the median values reported across the two sub-studies of the SHIP cohort (Male: 25.5–29.3 ml/kg/min; Female: 21.4–23.2 ml/kg/min) [24]. This comparison demonstrates that our research was conducted in a young cohort with superior CRF, higher metabolic resilience, and greater age homogeneity. Therefore, the profound sex dimorphism observed in postexercise lipid dynamics, even after controlling for age heterogeneity.

Compared to the pooled-sex analysis, a sex-stratified analysis identified a greater number of exercise-induced lipid changes (Figure 2A-C), directly underscoring the critical importance of considering sex in lipidomics research. In terms of overall trends, as time progressed postexercise, the pattern of altered lipids shifted toward downregulation.

This study utilized a multi-strategy analytical approach to identify five significant markers, including PC 36:4, PE 36:4, PC 32:1, SM d18:1/25:0, and BMP 34:1, that indicate a sexual dimorphism in response following acute exhaustive exercise.

PC constitutes approximately 40–50% of total phospholipids in mammalian biological membranes [25]. Since PC functions as a primary maintainer of membrane structural integrity and fluidity, its dynamic alterations profoundly reflect cellular structural responses to physiological stress [26]. PC 36:4 and PE 36:4 are enriched with arachidonic acid (AA), a critical polyunsaturated fatty acid (PUFA) and primary precursor for eicosanoids that regulate inflammatory responses, tissue repair, and vascular function [27–30]. Consequently, the observed fluctuations in these serum lipids reflect structural remodeling within the phospholipid pool. Since these lipids contain arachidonic acid, their mobilization increases the availability of substrates that could theoretically be utilized for eicosanoid synthesis. However, as inflammatory markers were not directly measured in this study, these lipid changes should be interpreted as a shift in the metabolic potential for inflammation rather than direct evidence of an active inflammatory response. Our identification of AA-rich phospholipids (e.g., PE 36:4, PC 36:4) as key sex-discriminating markers aligns with the established baseline sexual dimorphism in phospholipid biosynthesis. Literature indicates that estrogen upregulates the Phosphatidylethanolamine N-methyltransferase-Fatty Acid Desaturases (PEMT-FADS) axis, priming the female liver for efficient synthesis of long-chain PUFA-containing phospholipids [31]. This higher baseline enzymatic capacity likely enables females to mobilize these bioactive lipid precursors more effectively for membrane remodeling and signaling modulation in response to acute exercise stress, contrasting with the male profile. Alterations in PC 32:1 are of particular interest as this lipid serves as a carrier for palmitoleic acid (16:1), a lipokine known to modulate insulin signaling [32–34]. Consequently, the observed sex-specific dynamics of PC 32:1 suggest a potential avenue for future research regarding how lipid remodeling might differentially interact with postexercise insulin sensitivity in males and females.

The very-long-chain SM d18:1/25:0 is a key participant in the formation of stable cell membrane domains [35], which are critical for overcoming the immense physical and oxidative stress induced by acute exhaustive exercise [36, 37]. The contribution of SM d18:1/25:0 to the observed sex differences suggests distinct strategies for maintaining membrane physicochemical stability against the mechanical and oxidative shear stress induced by exhaustive exercise. These molecular-level differences indicate that exercise-induced sexual dimorphism is deeply embedded in multiple fundamental biological processes, including lipid mediator generation, signal transduction, and cellular structural maintenance. These specific molecules are important candidates for future research into the mechanisms of these sex differences.

By analyzing temporal patterns, our findings reveal distinct sexspecific metabolic strategies following acute exhaustive exercise. The male-dominant “sustained downregulation” pattern (Cluster 3) featured shorter-chain, saturated TAGs. This profile is consistent with the baseline tendency of males to accumulate visceral fat, which typically releases saturated fatty acids directly into the portal circulation for hepatic TAG synthesis [38]. This specific molecular profile points to a metabolic strategy prioritized for the rapid and efficient oxidation of these lipids for energy [3]. In contrast, the female-dominant “Elevated then Recovered” pattern (Cluster 2) involved a transient increase in more structurally complex TAGs. This phenomenon suggests a different metabolic priority, potentially reflecting a distinct postexercise lipid turnover capability involving hepatic very low-density lipoprotein (VLDL)-TAG handling and peripheral clearance [39, 40]. This process is plausibly influenced by the hormonal milieu of the luteal phase, as high estrogen levels are known to modulate lipoprotein lipase (LPL) activity in adipose tissue [41, 42], a key factor in TAG clearance.

This study has several limitations that should be acknowledged. First, its cross-sectional design establishes only correlations, not causality, and the use of serum cannot fully capture tissue-specific metabolic events. Second, while we controlled for the menstrual cycle, the lack of direct sex hormone measurements precluded more precise modeling of their influence. Significant inter-individual variability in hormone levels can exist even within the same menstrual phase, and direct hormonal measurements would have enabled more precise modeling. Methodologically, nontargeted lipidomics provides relative rather than absolute concentrations. The lack of absolute quantification makes it difficult to assess the actual physiological magnitude of these lipid changes, limiting direct comparisons with other studies and challenging the future establishment of functional thresholds or clinical intervention targets. The use of separate venipunctures precluded sampling immediately at exhaustion. Despite a strict 2-minute window, this brief delay may have missed biomarkers with rapid kinetics and introduced potential variability. Furthermore, the short 30-minute observation window and the specific cohort of healthy East Asian youth warrant caution when generalizing our findings. Finally, while we strictly standardized the final meal prior to testing to minimize acute postprandial variability, we did not control for habitual dietary intake. Sex-specific differences in habitual macronutrient preference could theoretically contribute to the observed baseline lipidomic dimorphism. Chronic manipulations of energy availability and macronutrient intake can profoundly alter the serum cardiometabolic profile, including lipoprotein subfractions and triglyceride concentrations [43]. In the context of their findings, our participants’ baseline status corresponds to the ‘energy-replete’ state (similar to their PREdiet phase), distinct from the metabolic alterations induced by low energy availability. This suggests that the sex differences observed in our study reflect intrinsic physiological dimorphism in an energy-balanced state, rather than adaptations to chronic energy restriction.

## CONCLUSIONS

This study reveals metabolic sexual dimorphism and lipid remodeling patterns when males and females reach standardized physiological exhaustion endpoints. Sex-stratified analysis identified 179 male-responsive and 288 female-responsive lipids, detecting 8.2%-115.3% more responsive lipids than pooled-sex analysis. Five sex-discriminating lipid molecular markers were identified (PE 36:4, PC 36:4, PC 32:1, SM d18:1/25:0, and BMP 34:1). Cluster analysis revealed three recovery patterns, with lipid distribution differing significantly by sex. These findings underscore the necessity of sex-stratified approaches for accurate exercise biomarker interpretation, personalized training protocol development, and targeted metabolic intervention implementation, providing a molecular foundation for precision medicine translation in exercise physiology.

## Supplementary Material

Sex dimorphism in serum lipid dynamics after acute exhaustive exercise
